# The influence of bio-based monomers on the structure and thermal properties of polyurethanes

**DOI:** 10.1038/s41598-024-80358-6

**Published:** 2024-11-23

**Authors:** Joanna Brzoska, Janusz Datta, Rafał Konefał, Václav Pokorný, Hynek Beneš

**Affiliations:** 1 Faculty of Chemistry, Department of Polymer Technology, Gdansk University of Technology, 11/12 Gabriela Narutowicza Street, Gdansk, 80-233 Poland; 2 Institute of Macromolecular Chemistry, Czech Academy of Sciences, Heyrovského nám. 2, Prague, 162 00 Czech Republic

**Keywords:** Sustainable development, Climate change, Natural resource, Biomass, Polyurethane, Climate sciences, Chemistry, Materials science

## Abstract

Most polyurethanes (PU) are currently produced through the polyaddition reaction of polyisocyanates with polyols and chain extenders, using components of petrochemical origin. From an environmental and geopolitical point of view, and with regard to the problems of oil supply and processing, the replacement of petrochemical PU raw materials with renewable resources is highly desirable. It is also one of the principles of sustainable development and an important challenge for chemical companies and market competitiveness. Current research studies focus mainly on the use of bio-based polyols for PUs, while other PU components, in particular polyisocyanates, remain of petrochemical origin. In this work, a series of PUs have been synthesized by polyaddition reactions of different types of renewable polyols and bio-based polyisocyanates. The effects of the bio-derived components on the structure, thermal stability and phase transformations of the PU were studied using FTIR and NMR spectroscopy, SWAXS, TGA, DSC, DMTA and TGA-FTIR. A full conversion of the bio-based monomers was achieved in all cases, indicating good compatibility and reactivity of all bio-based components. It was observed that bio-based PU exhibited a lower degree of phase separation and slightly lower thermal stability compared to PUs from petrochemical monomers.

##  Introduction

Commercial, polyurethanes (PU)s are obtained by the polyaddition reaction of di- or polyisocyanates with polyols and chain extenders^1–3^. Polyols and isocyanates are the main components of the formulation, which significantly determine the properties of the PU materials^4,5^. The amount of isocyanate in the formulation is generally around 30–40 parts by weight. The isocyanate-derived parts of PU structure can form hard segments (HS), which are responsible for the hardness, mechanical strength, and thermal stability of PU materials^6–8^. Unfortunately, the commercial-available isocyanates are of petrochemical origin, obtained by the reaction of amines with toxic phosgene. Isocyanate synthesis is highly unfavorable, dangerous to human health and not environmentally friendly^7,9,10^. Therefore, research has been carried out to find alternative and more environmentally friendly solutions to produce isocyanates using a safer synthesis route and non-petrochemical raw materials^11^.

Up to now, only a few bio-based polyisocyanates have been described in the literature^12–14^. Commercially available polyisocyanates are mainly derived from plants (fatty acids). Currently, there are three main types of bio-based isocyanates on the market: 1,5-penthamethylene diisocyanate (PDI) and its oligomers; L-lysine ethyl ester diisocyanate (LDI)^15^; and diisocyanate derived from 1,6-hexamethylene diisocyanate (HDI) and palm oil^16^. Covestro produces bio-derived PDI from plant sugars using a synthesis process that significantly reduces energy consumption. The PDI has a green carbon content of over 71% and although this bio-based monomer is not commercially available, it is further used to synthesize an aliphatic PDI trimer. This trimer is available on the market under the trade name DESMODUR^®^ eco N 7300^17^ and is dedicated to the synthesis of PU coating systems that are resistant to light, weather, and scratches^17^.

Aliphatic polyisocyanurates based on bio-based 1,5-pentamethylene diisocyanate is produced by Mitsui Chemicals under the trade name STABIO™ PDI. The offered monomer is available in two variants with low and high viscosity. The company recommends to use it for the synthesis of PU-urea materials^18^.

Another bio-based diisocyanate producer is Vencorex Chemicals, which offers an aliphatic bio-monomer under the trade name Tolonate™ X FLO 100. It is obtained from vegetable oil derivatives with a green carbon content of up to 32%. This monomer is used for the synthesis of elastomers and PU coatings^16^. As the exact chemical formula of this diisocyanate was not disclosed by the manufacturer, the authors of the publication “Fully Bio-Based Thermosetting Polyurethanes from Bio-Based Polyols and Isocyanates” proposed its structure based on electrospray ionisation mass spectrometry (ESI-MS negative)^19^. Based on this, Tolonate™ X FLO 100 contains a long-chain side group (labelled RO-) derived from vegetable oils. This complicates the synthesis of PUs due to the presence of additional functional groups and double bonds in the RO- labeled bio-diisocyanate fragment, resulting in additional cross-linking of the material.

The raw materials market for the synthesis of PUs also includes L-lysine ethyl ester bio-diisocyanate (LDI)^20^. It is produced from the amino acid L-lysine extracted from plant biomass. The LDI-based PUs exhibit low toxicity and biodegradability^21^.

Furthermore, companies like General Mills Inc^22,23^. and BASF Europe^24^ have developed isocyanates derived from fatty acid dimers. These innovative compounds have found applications in multiple industries, particularly in the production of ultraviolet-curable coatings, where they offer enhanced performance and sustainability.

It is well known that some applications require PUs to have special properties, including resistance to very high temperatures. For example, high-temperature resistant adhesives are required in advanced aircraft, rockets, and ground vehicles^25^. Therefore, an important parameter determined in the analysis of PU materials is their thermal stability, which describes the thermal durability of PU^26^. Polymers with higher thermal stability have higher melting, softening, and thermal decomposition temperatures, lower weight loss during heating at high temperatures, without losing their basic properties^27^. Various tests are carried out to evaluate these properties, including thermogravimetric analysis (TGA) or differential scanning calorimetry (DSC). This paper focuses on a detailed analysis of the structure and thermal stability of the obtained bio-based PUs as promising alternatives to the current petrochemical PUs.

In this work, renewable monomers were used to synthesize bio-based PU by polyaddition reaction. The reference PU materials were prepared from the petrochemical polyols and hexamethylene diisocyanate, using 1,3-propanediol as a chain extender. Meanwhile, the preparation of polyurethanes based on bio-based monomers with desired thermal properties remains an open challenge. Here, we describe the synthesis and characterization of novel polyurethanes based on bio-based PO3G (polyether polyol), semi-crystalline, bio-based polyester polyol (Priplast 3294), and partially bio-based isocyanate Tolonate X FLO 100 with bio-based chain extender 1,3-propanediol. Fourier Transform Infrared (FTIR) spectroscopy, Nuclear Magnetic Resonance (NMR) and Small and wide-angle X-ray scattering (SWAXS) were used to characterize all samples. The thermal properties of PUs were studied by TGA, DSC, DMTA and combination of FTIR spectroscopy and thermogravimetric analysis (TG-FTIR).

## Experimental

### Materials

For reference samples, two types of polyols were used: polyether polyol - poly(tetramethylene glycol) (PTMG) supplied by Overlack (Ozorków, Poland) and polyester polyol - Polios 55/20 purchased from Purinova (Bydgoszcz Poland) both with a molecular weight of 2000 g/mol. Hexamethylene diisocyanate (HDI), 1,3-propanediol (PDO) and catalyst dibutyltin dilaurate were purchased from Sigma Aldrich (Saint Louis, USA) and used as received. The reaction inhibitor was orthophosphoric acid supplied by POCH Gliwice (Gliwice, Poland).

For the synthesis based on renewable raw materials we used aliphatic isocyanate Tolonate X FLO 100 purchased from Vencorex (Saint-Priest, France) and two types of polyols: polyester polyol - PRIPLAST 3294™ purchased from Croda (Snaith, UK) and polyether polyol - Velvetol H2000 supplied by Allessa GmbH (Frankfurt, Germany) both with a molecular weight of 2000 g/mol. The chain extender Zemea (1,3-propanediol) was purchased from DuPont Tate & Lyle Bio Product (Naucalpan de Juárez, USA). The same catalyst and inhibitor were used as for the reference samples. The chemicals were used without further purification unless stated otherwise.

### Synthesis of PU elastomers

**Fig. 1:**
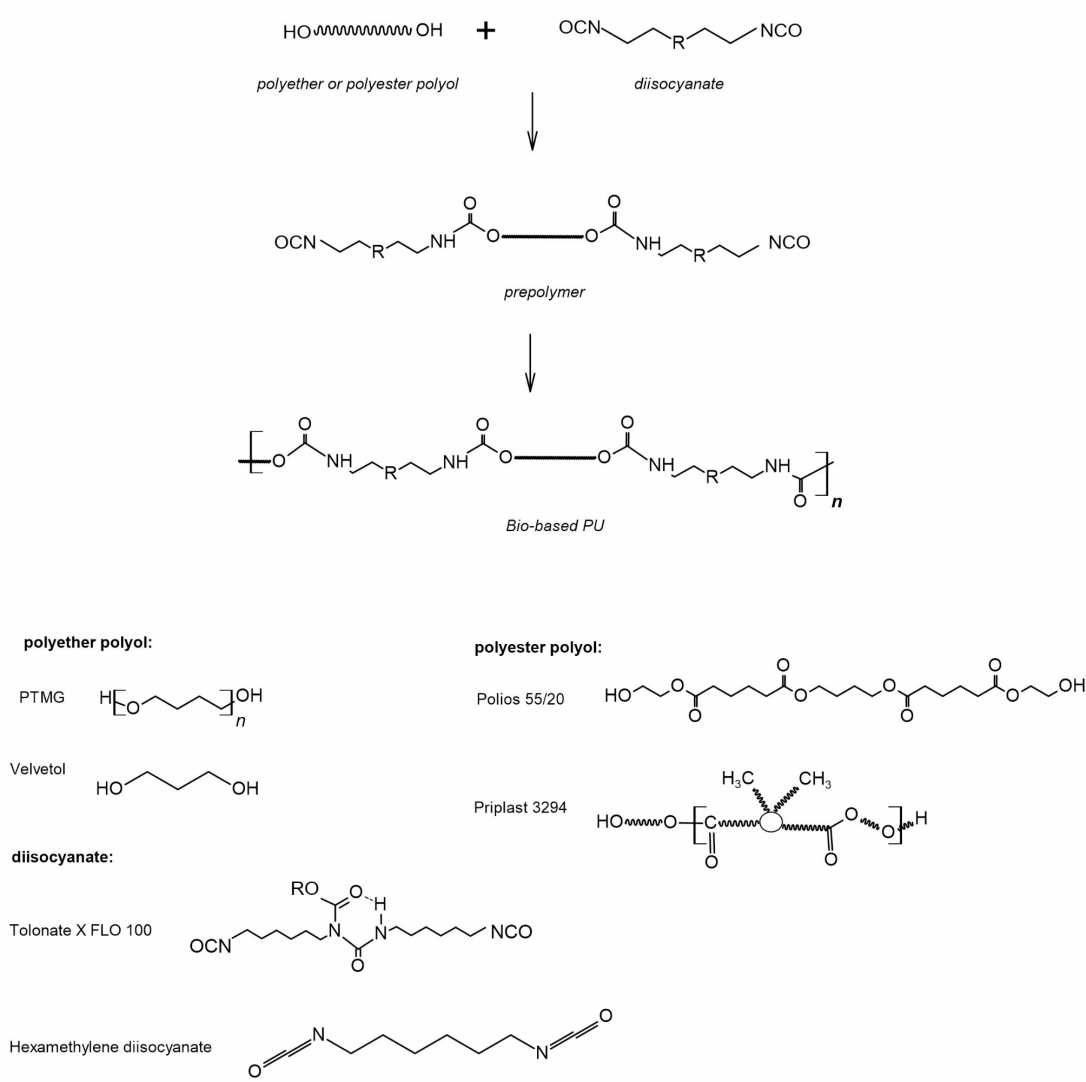
Reaction scheme for ether- and ester-urethane prepolymers formation.

The PUs were obtained using a two-step prepolymer method. The first step was to synthesize the prepolymer by reacting the polyol with an excess of diisocyanate at 85 °C for 2 h under vacuum. During the reaction, the percentage of NCO groups were determined by titration according to ISO 14896:2010 (isocyanate is reacted with an excess of n-dibutyl amine to form substituted ureas. The excess dibutyl amine is then back-titrated with aqueous hydrochloric acid. The solvent used is acetone). A chain extender and catalyst (0.1% DBTL) were then added to the prepolymer, and the mixture was stirred for 60 s. and degassed under vacuum for 60 s. The molar ratios of [NCO]/[OH] groups was 1.05. Finally, the materials were cured in a laboratory oven at 80-100 °C for 24 h. Figure 1 shows the overall method for the synthesis of ethers and ester-urethanes from bio-based monomers. Table 1 presents the formulation of the synthesized samples, the content of unreacted isocyanate groups in prepolymers (NCO content) and the content of HS. The content of bio-monomers (bio-content) was calculated using Eq. (1) and is given in Table 1.

1 $$\:bio-content=\:\frac{{M}_{BIO}}{{M}_{TOTAL}}\times\:100\boldsymbol{\%}$$

$\:{M}_{BIO}$ - the molar mass of the used bio-monomers. $\:{M}_{TOTAL}$ – the molar mass of all substrates used.

**Table 1:** Sample summary.

Sample code	Polyol	Isocyanate	Glycol	NCO content [%]	HS content [%]	Bio-based content in PU [%]
HPoP	Polios 55/20	HDI	PDO	7.8	27.5	0
HPTP	PTMG	HDI	PDO	7.7	27.4	0
TPobP	Polios 55/20	Tolonate X FLO 100	bio-PDO (Zemea)	7.5	41	23.8
TPTbP	PTMG	Tolonate X FLO 100	bio-PDO (Zemea)	7.7	41	23.8
TVbP	Velvetol H2000	Tolonate X FLO 100	bio-PDO (Zemea)	7.7	41	52
TPribP	Priplast 3294	Tolonate X FLO 100	bio-PDO (Zemea)	7.8	41	52

### Methods of characterization

#### Fourier transform infrared spectroscopy

The chemical structure of the bio-based PU was analyzed by Fourier Transform Infrared Spectroscopy using a Spectrum 100T FT-IR spectrometer with universal ATR accessory (PerkinElmer, USA). Each spectrum was recorded at room temperature for wavenumber range of 500 to 4500 cm^− 1^ with a resolution of 4 cm^− 1^.

The degree of the carbonyl groups participating in hydrogen bonding can be described by the carbonyl.

hydrogen bonding index, R, as given in Eq. (2).

2 $$\:R=\frac{{A}_{bonded}}{{A}_{free}}$$

where A is the intensity of the characteristic absorbance (A_H−bonded_ – absorption intensity of hydrogen-bonded carbonyl; A_H−free_ – absorption intensity of free carbonyl). The degree of phase separation (DPS) and the degree of phase mixing (DPM) can be obtained by using Eqs. (3) and (4), respectively.

3 $$\:DPS=\:\frac{R}{R+1}$$

4 $$\:DPM=1-DPS$$

### Nuclear magnetic resonance

^1^H NMR spectra of prepared solutions (DMSO or DMF) were acquired with the Bruker Avance III spectrometer operating at 600.2 MHz. The width of 90° pulse was 18 µs, relaxation delay 10 s, acquisition time 2.73 s, 32 scans.

### Small and wide-angle X-ray scattering

The internal microstructure of PU samples was investigated through Small-Angle and Wide-Angle X-ray Scattering (SAXS and WAXS) analysis. The measurements were conducted using a pinhole camera (MolMet, Rigaku, Japan, modified by SAXSLAB/Xenocs) connected to a microfocused X-ray beam generator (Rigaku MicroMax 003) operating at 50 kV and 0.6 mA (30 W). Scattering intensities were recorded using a vacuum-compatible hybrid photon-counting detector (Pilatus3 R 300 K), with a sample-to-detector distance of 1000 mm (SAXS) and 50 mm (WAXS), respectively, using an exposure time of 3 h.

### Thermogravimetric analysis

TGA of the PUs were performed using a Pyris 1 TGA Thermogravimetric Analyzer (PerkinElmer, USA). Approximately 10 mg of the sample was placed in a ceramic crucible and heated from 35 to 600 °C at a heating rate of 10 °C/min under a nitrogen flow of 20 mL/min. The standard deviation of the TGA measurement was under 5%.

### Differential scanning calorimetry

The thermal behaviour of the polyurethanes was evaluated by differential scanning calorimetry (DSC) using a TG 209F1 Libra (NETZSCH, Germany). The samples (5–10 mg) were encapsulated in aluminium hermetic pans. The measurements were carried out in a heating-cooling-heating cycle at the temperature range of (-80) – 250 °C. The analyses were performed at a heating rate of 10 °C/min in an inert (nitrogen) atmosphere.

### Thermogravimetric analysis coupled with Fourier transform infrared spectroscopy

Thermogravimetric analysis coupled with Fourier transform infrared spectroscopy (TG-FTIR) was carried out using a Netzsch TG 209 thermal analyser (heating rate = 10 K/min, sample mass 10–15 mg, nitrogen flow = 20 ml/min) and a Bruker Alpha FTIR spectrometer. IR spectra were recorded in the spectral range of 4000–500 cm^− 1^ with a 4 cm^− 1^ resolution and 8 scans.

### Dynamic mechanical and thermal analysis

Dynamic-mechanical and thermal analysis (DMTA) of the prepared PU samples was measured on an ARES G2 rheometer (TA Instruments). Oscillatory shear deformation (0.02–1% strain) at a frequency of 1 Hz from − 100 °C to 150 °C at a temperature ramp rate of 3 °C/min was applied on rectangular samples (20 × 5 × 2 mm). The temperature dependence of the complex shear modulus (G*) was observed and the main transition temperature (*T*α) was determined to be the tan δ peak maximum.

## Results and discussion

### Fourier transform infrared spectroscopy

**Fig. 2:**
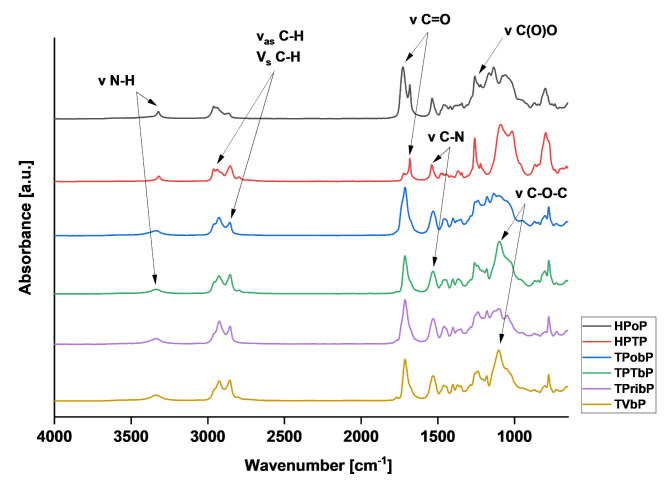
FTIR spectra of PUs.

The chemical structure of the PUs was confirmed by FTIR-ATR and ^1^H NMR spectroscopy. The FTIR spectra are shown in Fig. 2 and the characteristic bands are given in Table 2. The characteristic isocyanate (-N = C = O) and polyol (-OH) bands at 2260 cm^− 1^ and 3600 cm^− 1^, respectively, were not observed in any of the samples^28^. Meanwhile, the bands corresponding to the urethane groups in the range of 3400–3300 cm^− 1^ and the I amide band in the range of 1740–1680 cm^− 1^ characteristic for the vibration of the -C = O group, confirm the full rearrangement of substrates^29,30^. At 1528–1540 cm^− 1^ two vibrations were assigned to the urethane CN-H group part: a bending vibration for N–H and a stretching vibration for C–N. The band at 2937–2850 cm^− 1^ can be assigned to symmetric and asymmetric CH stretching vibrations originating from the CH_2_ groups present in aliphatic chains, or from the CH_3_ groups. The next bands are: stretching vibrations of C–O–C in ether bonds at 1160 –1060 cm^− 1^ and in ester bonds at 1250 –1150 cm^− 1^.

The graphs show no significant differences in structure between the reference samples and bio-PUs, confirming the possibility of obtaining PU from bio-based monomers.

**Table 2:** Characterization of selected FTIR-ATR bands of the obtained PUs.

Wavenumber [cm^− 1^]	Assignments
3338–3322	-NH stretching
2933–2850	-CH_2_ symmetrical and asymmetrical
1726–1681	-C = O stretching
1540–1528	-C-N stretch
1250–1150	Stretching vibrations of C(O)O in ester bonds
1160–1060	Stretching vibrations of C–O–C in ether bonds

The obtained values of R, DPS and DPM for PU were given in Table 3. The DPS value describes the contribution of HS bonded among themselves by hydrogen bonds, while the DPM value describes the contribution of HS not bonded by hydrogen bonds to any other HS^31^. As has been previously reported in the literature, the DPS value in PUs increases with increasing R^32^. The HPTP sample has the highest value of R and therefore shows the highest phase separation compared to other samples. It was confirmed that the content of HS in the PU structure and the origin of the substrates used in the synthesis affect the DPS value. It was observed that as the HS content increases, DPS decreases. This is related to the fact that materials with a higher content of HS have more urethane groups, which can bond with the ester groups of polyols, increasing interactions between HS and SS and resulting in phase mixing. It was also observed that materials obtained from petrochemical monomers have higher DPS contents. This is in line with reports from other authors who also observed that the highest DPS value was recorded for HDI-based materials. This may be related to the linear structure of this isocyanate^33^. Moreover, differences were observed due to the nature of the polyol used. PUs based on polyether polyols were characterized by higher DPS values compared to PUs based on polyester polyols. For samples containing branched HS, i.e. based on bio-isocyanate, a decrease in DPS values was observed, probably due to the higher free volume between macromolecules, which hinders the formation of hydrogen bonds.

**Table 3:** The carbonyl hydrogen bonding index (R), the degree of the phase separation (DPS), and the degree of the phase mixing (DPM) in the obtained PUs.

Sample	A_bonded_^*^ (ν [cm^− 1^])	A_free_^**^( ν [cm^− 1^])	*R*	DPS	DPM
HPTP	0.68311 (1681)	0.34143 (1695)	2.0	0.67	0.33
TPTbP	0.30617 (1692)	0.80399 (1714)	0.38	0.275	0.725
TVbP	0.29631 (1691)	0.81993 (1714)	0.361	0.265	0.735
HPoP	0.44942 (1683)	0.97873 (1726)	0.459	0.315	0.685
TPobP	0.15097 (1670)	0.91303 (1716)	0.165	0.142	0.858
TPribP	0.18518 (1673)	0.92041 (1713)	0.201	0.167	0.833

A: absorption intensity calculated as the area of Gaussian multipeak fitting; * A_free_: absorption intensity of free carbonyl. ** A_bonded_: absorption intensity of hydrogen-bonded carbonyl.

### Nuclear magnetic resonance

^1^H NMR spectra with a description of signals of the samples dissolved in DMSO and DMF are presented in Table 4. The type of solvent was determined by the solubility of the materials tested (due to the limited solubility of the TPTbP and TPribP samples, their spectra were prepared for prepolymers). The signals labeled a (in DMF), f (in DMSO) and k (TPribP in DMSO) in the range of 6.9–8.5 ppm are assigned to -NH in the urethane group. The peaks from 4.0 to 4.3 ppm indicate, hydrogen present in the main chain next to oxygen in the structure of this material. The range 1.2–1.7 corresponds to the protons of the methylene groups derived from the aliphatic chains of the polyol. The peaks in the range of 2.9–3.2 are related to the methylene bridge attached to the urethane nitrogen. FTIR and ^1^H NMR analyses confirmed the reaction of the -NCO groups of the diisocyanate with the -OH groups of the polyol used, as a signal characteristic of the protons of the -NH group was observed.

**Table 4:**
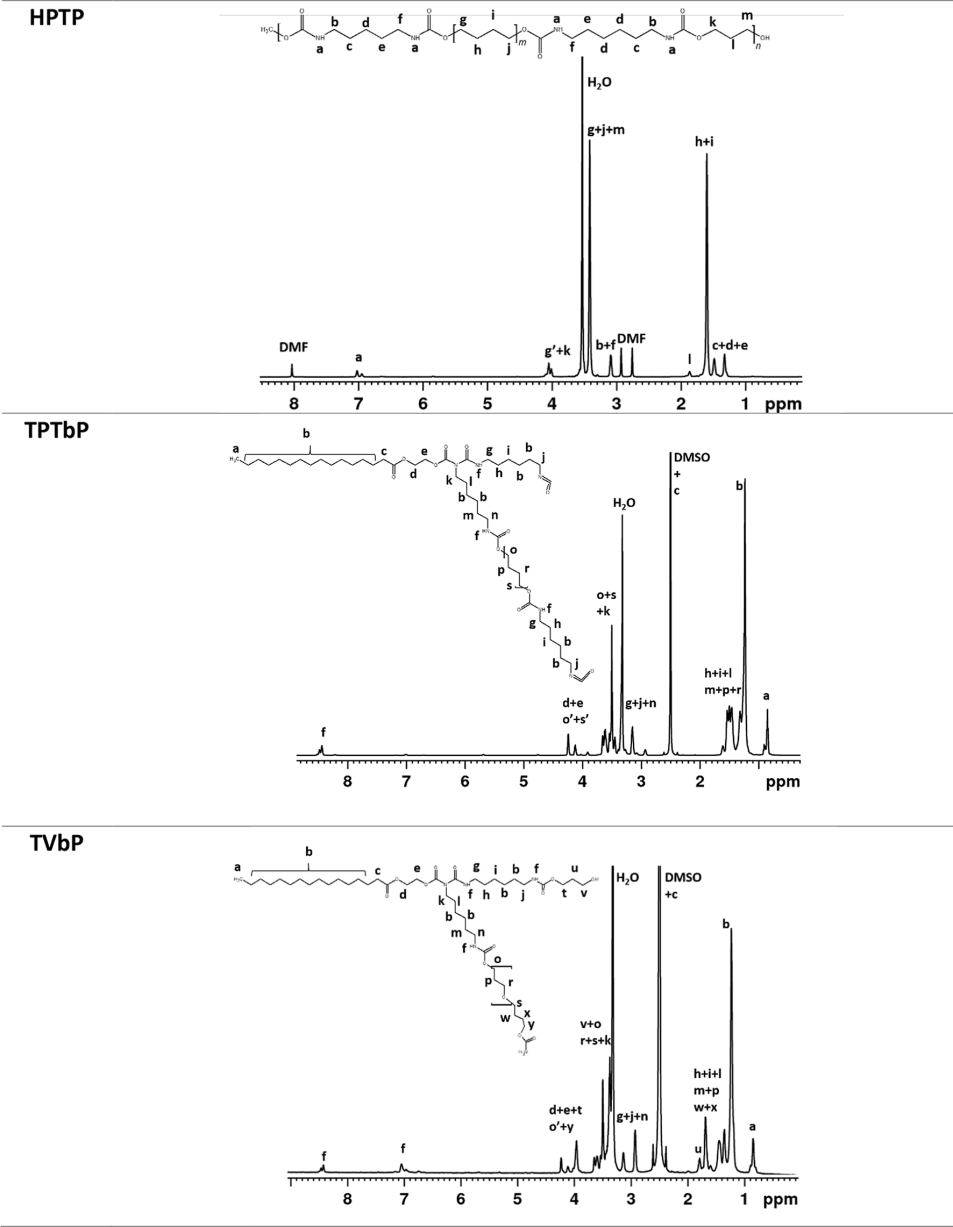

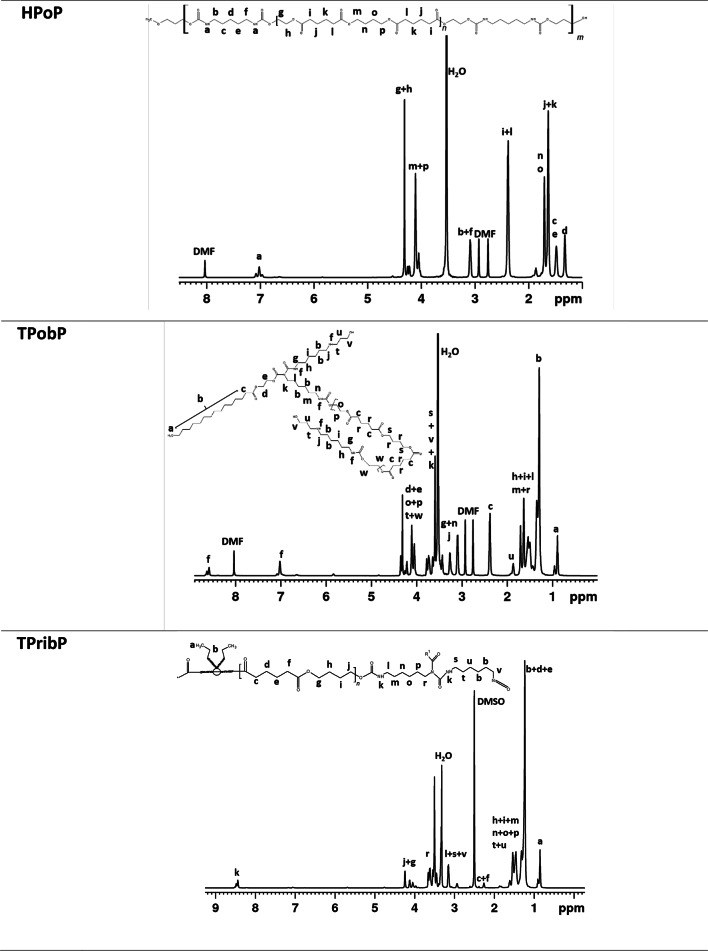
^1^H nuclear magnetic resonance (NMR) spectra of the obtained samples in DMSO and DMF.

### X-ray scattering

**Fig. 3:**
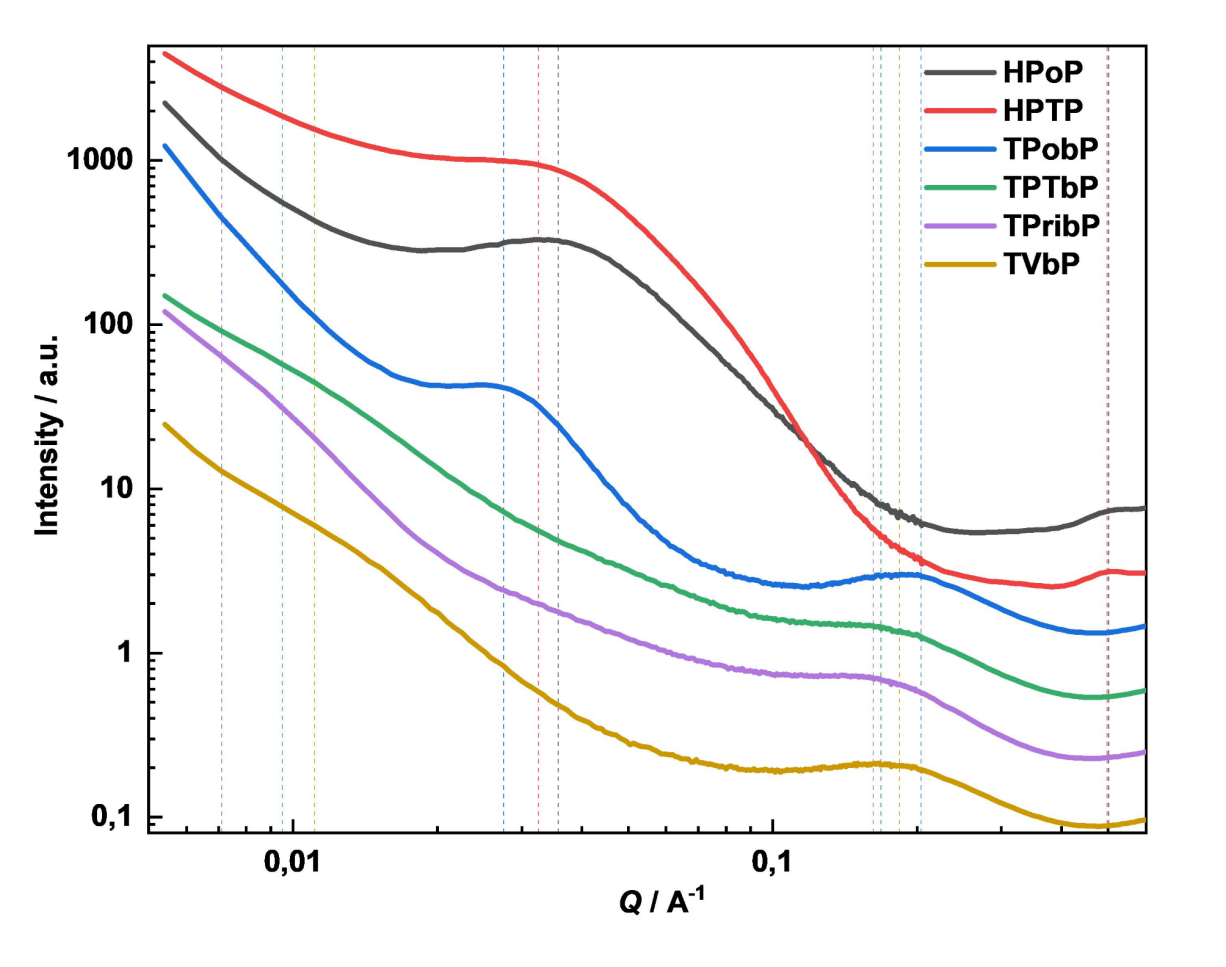
SAXS curves of PUs. Vertical lines indicate the positions of peaks.

All PU samples exhibit two distinct peaks in their SAXS profiles (Fig. 3), though the peak positions vary between samples. These peak positions, along with the corresponding characteristic sizes, are summarized in Table 5. The peak at lower Q-values is attributed to the microphase separation between the soft and hard segments, while the higher-Q peak corresponds to the periodic structure within the hard segment domains.

For samples containing HDI-based isocyanate, both peaks shift to higher Q-values, indicating a more compact or tightly packed structure, with smaller domain sizes. Additionally, the reduced intensity of the second peak suggests a lower hard segment (HS) content or reduced crystallinity, reflecting fewer or less well-defined hard segment domains.

**Table 5:** SAXS peak positions and corresponding sizes of structural elements.

Sample code	Peak 1 – microphase separation		Peak 2 – structure of hard segments	
	Q/A^− 1^	d/nm	Q/A^− 1^	d/nm
HPoP	0.0357	17.6	0.502	1.3
HPTP	0.0325	19.3	0.498	1.3
TPobP	0.0275	22.8	0.204	3.1
TPTbP	0.0095	66.1	0.168	3.7
TPribP	0.0071	89.1	0.162	3.9
TVbP	0.0111	56.6	0.184	3.4

**Fig. 4:**
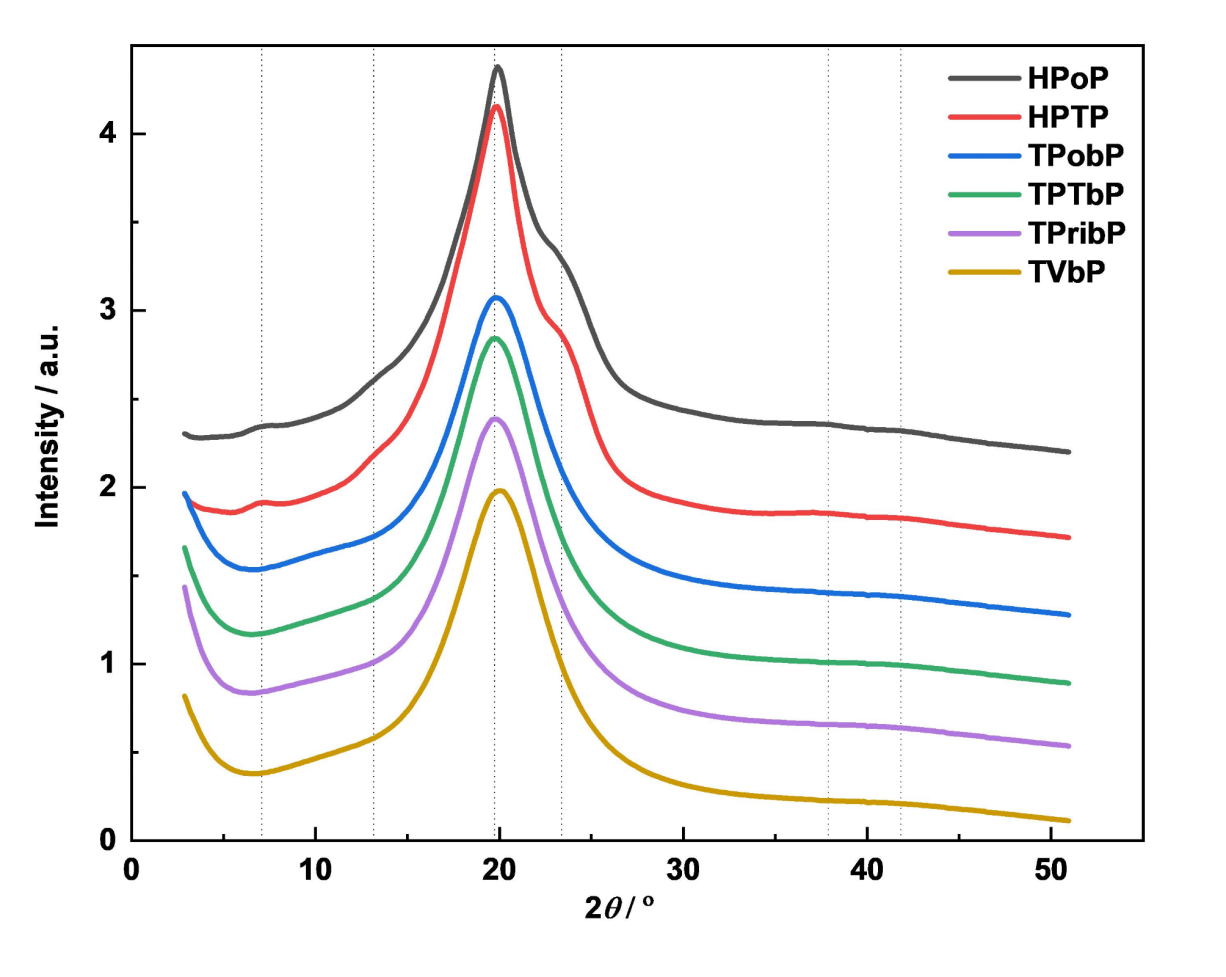
WAXS curves of PUs. Vertical lines indicate the positions of peaks.

The Wide-Angle X-ray Scattering (WAXS) curves (Fig. 4) reveal differences in short-range ordering between the HDI-based and Tolonate-based PU samples. These differences are likely due to variations in polymer chain arrangement and crystalline domain structure. The HDI-based samples exhibit several additional diffraction peaks, suggesting the presence of more complex or diverse structural motifs compared to the Tolonate-based PUs.

### Differential scanning calorimetry

**Fig. 5:**
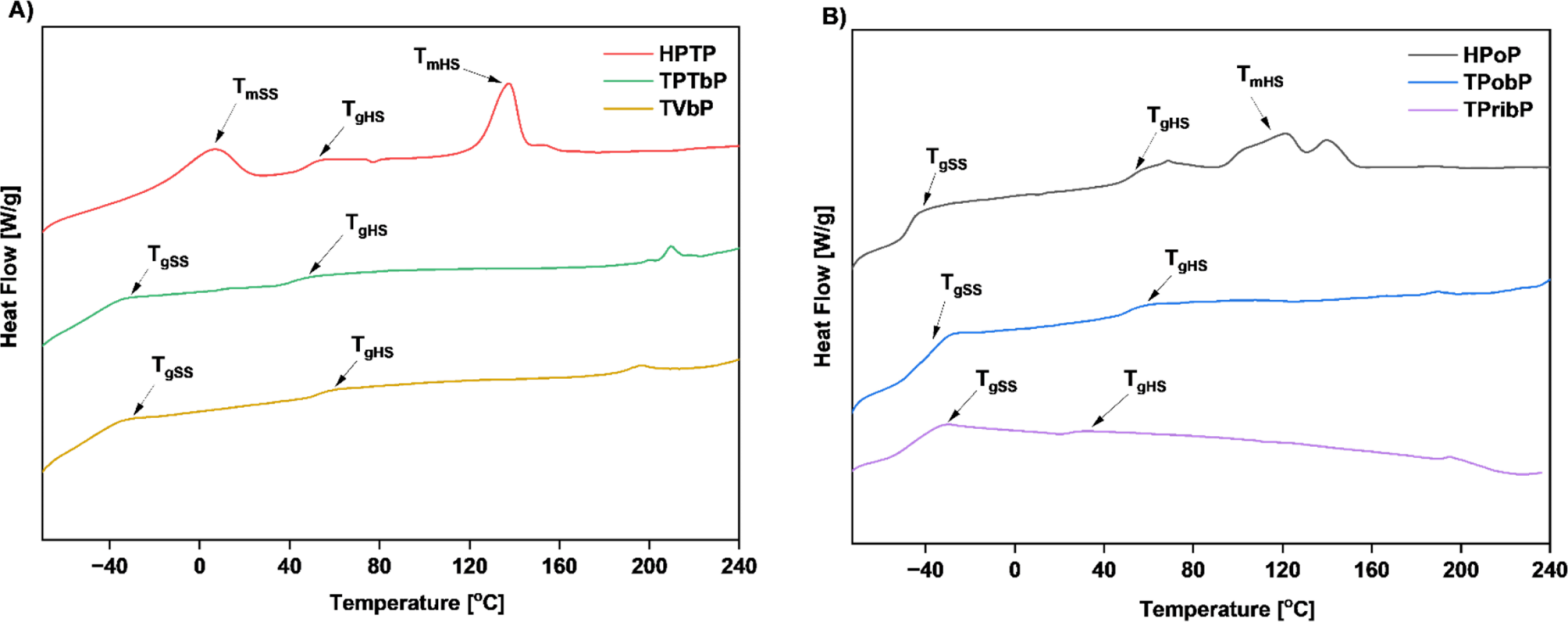
DSC curves (first heating) of the obtained PUs: (**A**) based on polyether polyol (**B**) based on polyester polyol.

**Fig. 6:**
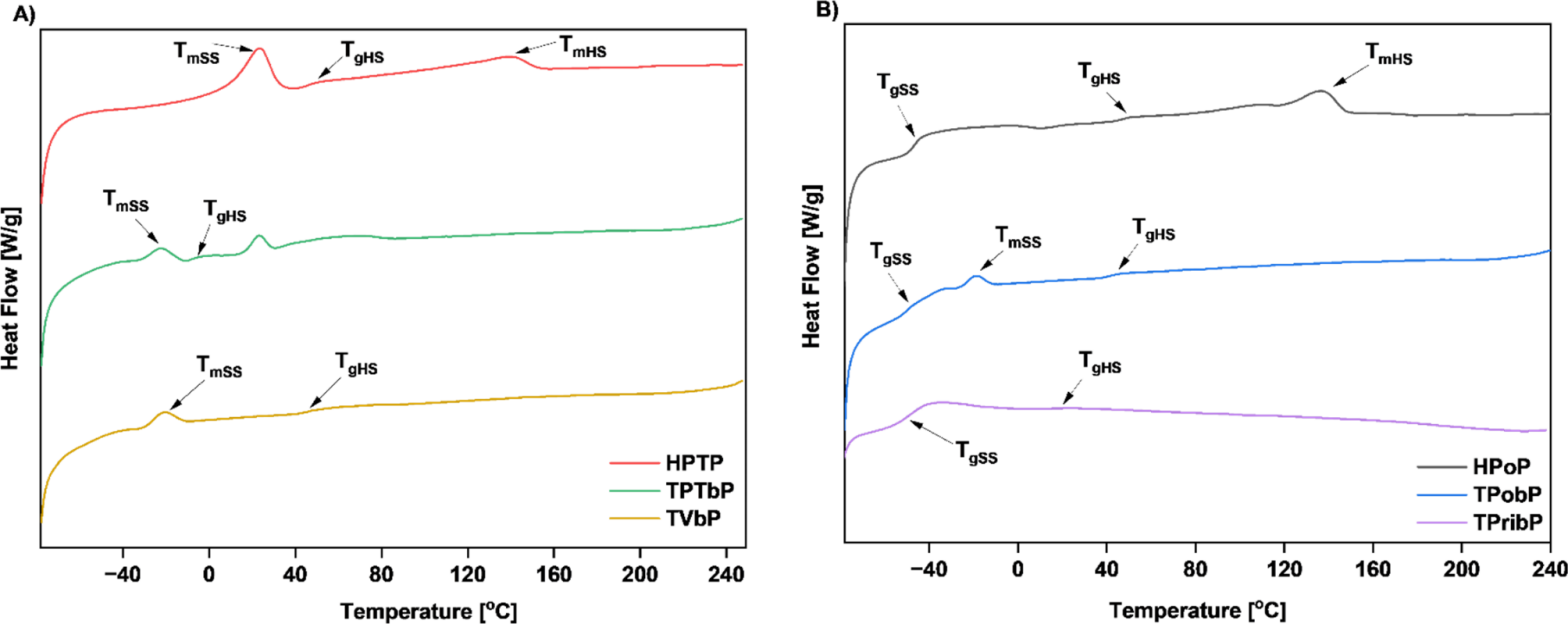
DSC curves (second heating) of the obtained PUs: (**A**) based on polyether polyol (**B**) based on polyester polyol.

The glass transition temperature (T_g_) is an important characteristic of polymeric materials because it represents the boundary between their elastic and viscoelastic behavior. Figures 5 and 6 present DSC thermograms obtained from DSC experiments during the first and second heating of PUs.

Thermal behavior of the obtained materials such as, glass transition temperature of SS (T_gSS_) and HS (T_gHS_), melting point of SS (T_mSS_) and HS (T_mHS_), and enthalpy are presented in Table 6. The T_g_ values mainly depended on the type of monomers. Glass transition temperatures for SS and HS and melting points for SS and HS were observed. During the first heating in the DSC, we observe melting temperatures for the reference samples, while no melting peaks are detected for the bio-based PU. This confirms our observations from the WAXS results, which show negligible crystallinity for the bio-PU, whereas the reference PU samples have a significantly higher crystalline content (17.5% for HPoP and 24.4% for HPTP). During the 2nd heating the glass transition temperature for SS is mainly observed for polyester based samples. The T_g_ of the soft segments ranged from − 47.4 °C to -55 °C. The lack of a distinct glass transition peak for polyether-based samples may be related to the morphological characteristics of these materials. The flexibility of the polyether-based segments could reduce the sharpness of the glass transition, making it difficult to observe a distinct T_g_ peak. On the other hand, melting of the soft segments is observed for the polyether-based samples and the TPobP (polyester-based) sample. This is related to the reorganization of the soft segment microdomains after the second heating cycle, as a result of the removal of the sample’s thermal history during the first heating^34^. The transition observed for TPTbP sample during the second heating cycle at 22.9 °C was attributed to some relaxation effects of the polymer chain. A melting of the hard segments is observed for the reference samples during 1st and 2nd heating. This may be related to the higher DPS value of these samples. Because HDI-based samples have a more ordered structure than Tolonate-based samples^28^.

**Table 6:** Thermal properties of the samples determined by DSC (2nd heating).

Sample code	Soft Phase T_gSS_/°C	Hard Phase T_gHS_/°C	Soft Phase Melting/°C	Hard Phase Melting/°C	ΔH/Jg^− 1^ (melting SS/ melting HS)
HPTP	-	46.9	23.1	141.8	25.96/4.89
TPTbP	-	-	-22	-	4.49/3.46
TVbP	-	45.4	-21.8	-	4.35/ -
HPoP	-47.4	46.9	-	136.5	9.09/ -
TPobP	-47.4	41.1	-19.3	-	2.35/ -
TPribP	-55	18.1	-	-	-

### Thermogravimetry

**Fig. 7:**
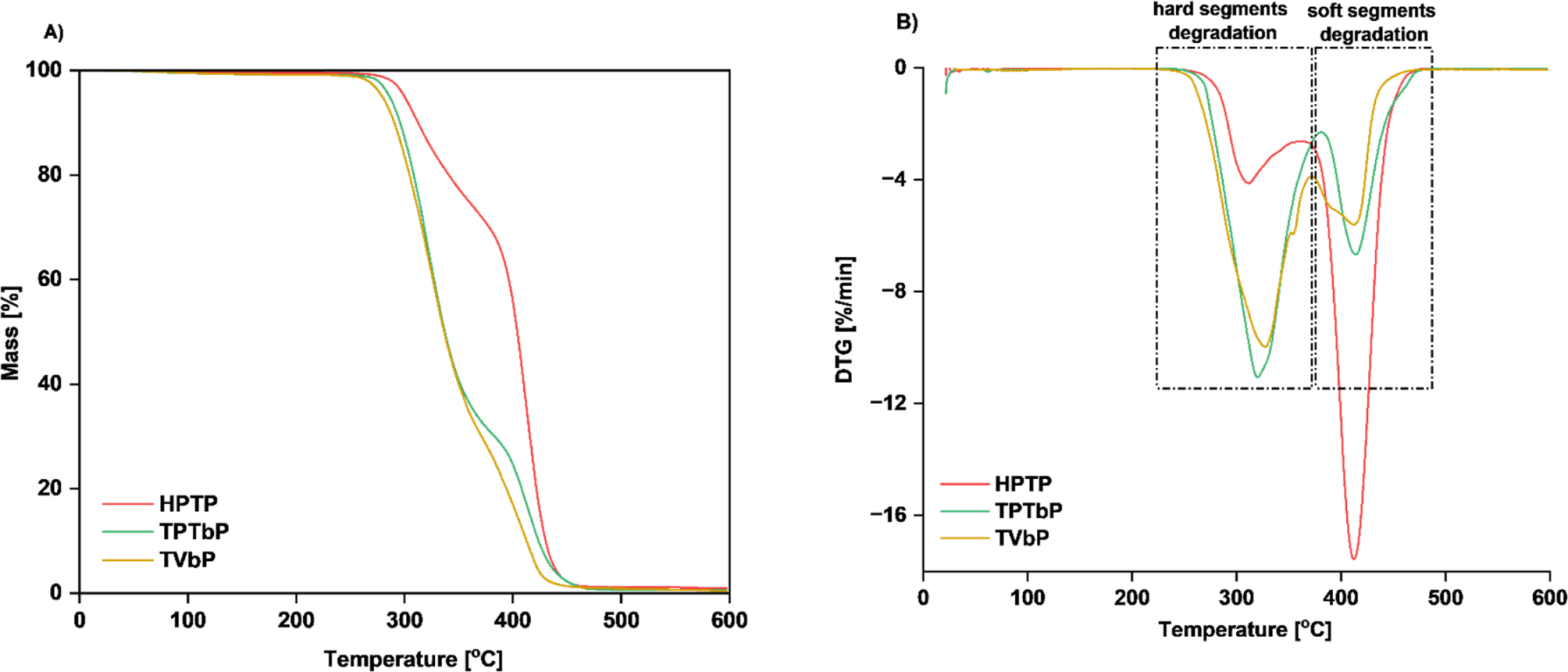
TG and DTG curves of polyether-based PUs. (**A**) TG; (**B**) DTG.

**Fig. 8:**
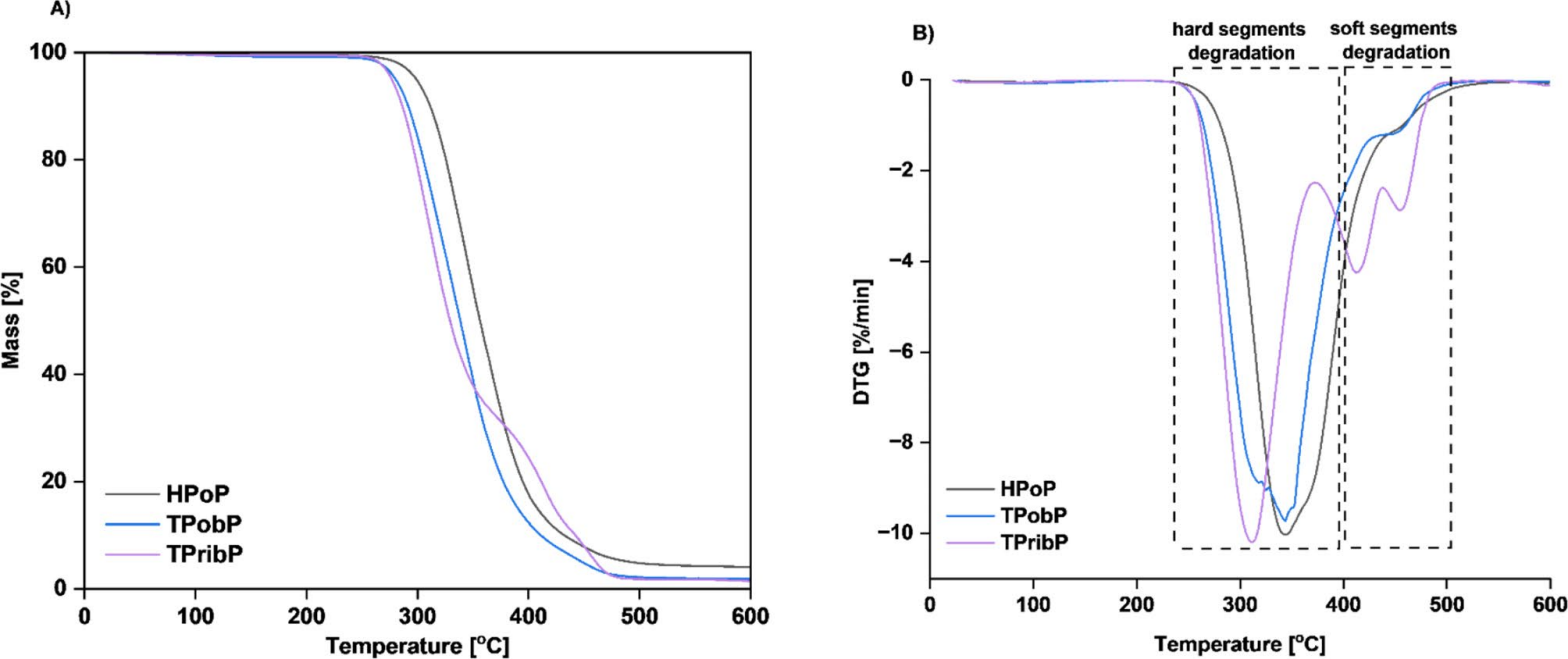
TG and DTG curves of polyester-based PUs. (**A**) TG; (**B**) DTG.

TGA is used to measure thermal decomposition and thermal stability of materials. The TGA measurements were carried out on PUs based on both bio-based monomers and petrochemical feedstocks. The aim was to investigate the thermal stability of the prepared materials and to determine the influence of the bio-based substrates on the final properties of the product. The TG and DTG curves are shown in Figs. 7 and 8. Table 7 shows the temperatures for 5%, 50% and 90% mass loss and the ash residue in mass % at 600 °C.

As it can be observed all samples exhibited two steps of degradation. The first step is attributed to the decomposition of HS, including mainly isocyanate residues and chain extenders. This is caused by the relatively low thermal stability of the urethane groups^35^. In contrast, the second step is attributed to the decomposition of SS, which is mainly composed of long polyol chains.

From the TG curves, it was observed that they are almost superposed, while the DTG curves are only partially overlapped. The bio-based PUs are slightly less stable than the reference samples because their initial decomposition temperature is lower (see Table 7).

**Table 7:** Temperature values at different mass losses [%].

Sample code	T_d5%_ [°C]	T_d50%_ [°C]	T_d90%_ [°C]	Char residue [%]
HPTP	297	403	429	0.94
TPTbP	284	336	423	0.53
TVbP	278	330	407	0.41
HPoP	295	355	426	4.0
TPobP	277	338	406	1.8
TPribP	274	328	437	1.38

T_d5%_ is the temperature of 5% mass loss, T_d50%_ is the temperature of 50% mass loss and T_d90%_ is the temperature of 90% mass loss.

The temperatures for the first step of mass loss are similar, ranging from 237 °C to 248 °C for samples based on polyester, and 238–251 °C for samples based on polyether. Therefore, we can conclude that polyether-based samples are more stable than those on polyester. This is related to the different structures of the polyols (symmetrical and short chain of Velvetol and PTMG, long chain of Polios, branched chain of Priplast). Furthermore, it can be observed that PUs derived from bio-isocyanates degraded at a lower temperature than those based on petrochemical HDI. For comparison, 5% weight loss of HPTP sample occurs at T = 297 °C, and TPTbP sample at T = 284 °C. This is related to the lower thermal stability of bio-based isocyanate compared to the stability of HDI. As the thermal stability of isocyanates is closely related to the symmetry of their structure^27^. When the symmetry of the isocyanate increases, the stability of the PU increases, so Tolonate X FLO 100 has a lower stability due to its branched structure. However, our Tolonate-based samples had only slightly lower thermal stability than those reported by other scientists, also based on Tolonate and Velvetol (with a different polyol mass, we used 2000 g/mol versus 500 g/mol), where the T_5%_ was 292 °C^19^.

### Thermogravimetric analysis coupled with Fourier transform infrared spectroscopy

The gases produced during TGA of the samples at different temperatures (50–800 °C) were analyzed by FTIR spectroscopy. Analysis of the spectra of volatile decomposition products confirmed the relationships presented in the TGA figures. Decomposition of HS occurs in the first step and, according to the literature, leads to the formation of isocyanate and al*c*ohol, primary or secondary amine, olefin, and carbon dioxide^8,36^. Meanwhile, in the second step, decomposition of SS consisting of polyols occurs. Temperatures in the range of 343–429 °C were chosen as the most representative points to illustrate characteristic groups of gas products.

**Fig. 9:**
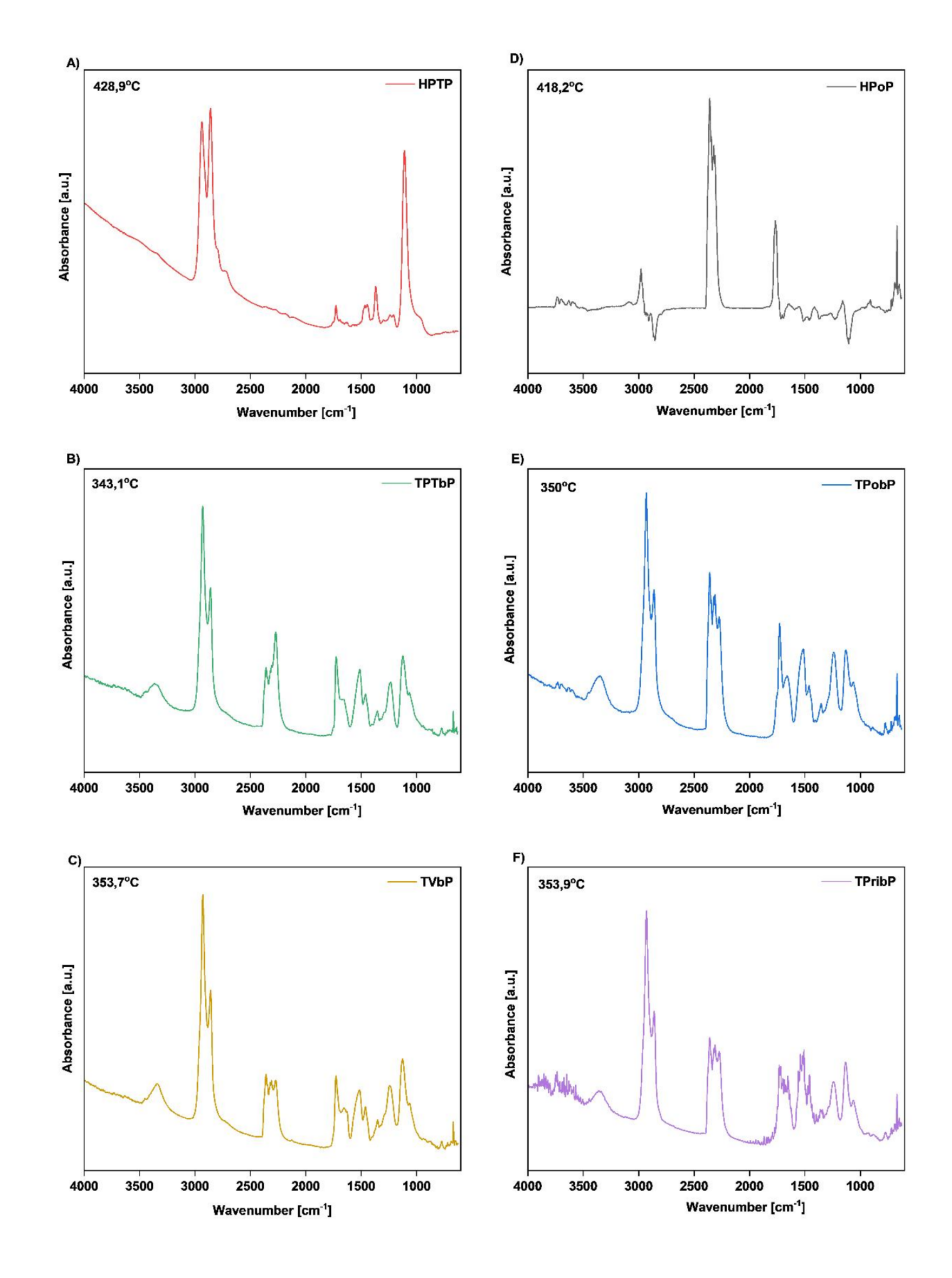
FTIR spectra of the obtained PU: (**A**) HPTP, (**B**) TPTbP, (**C**) TVbP, (**D**) HPoP, (**E**) TPobP, (**F**) TPribP at various temperature points.

All of the FTIR spectra shown in Fig. 9 exhibit absorption bands typical of carbon dioxide, at a wavenumber in the range 2391–2344 cm^− 1^, associated with asymmetric stretching vibrations, at 696–668 cm^− 1^, attributed to degenerate bending vibrations, and at 3598–3350 cm^− 1^ corresponding to combination bands. The spectrum also reveals bands at 2941–2928 cm^− 1^ and 2865–2849 cm^− 1^, characteristic of asymmetric and symmetric C-H stretching vibrations, and at 1479–1450 cm^− 1^, characteristic of asymmetric and symmetric C-H bending vibrations, of methylene and methyl groups.

The presence of a band at 1060–1067 cm^− 1^ confirms the formation of primary alcohols, associated with C-O stretching vibrations, and at 3649 –3626 cm^− 1^, associated with O-H stretching vibrations.

### Dynamic mechanical and thermal analysis

**Fig. 10:**
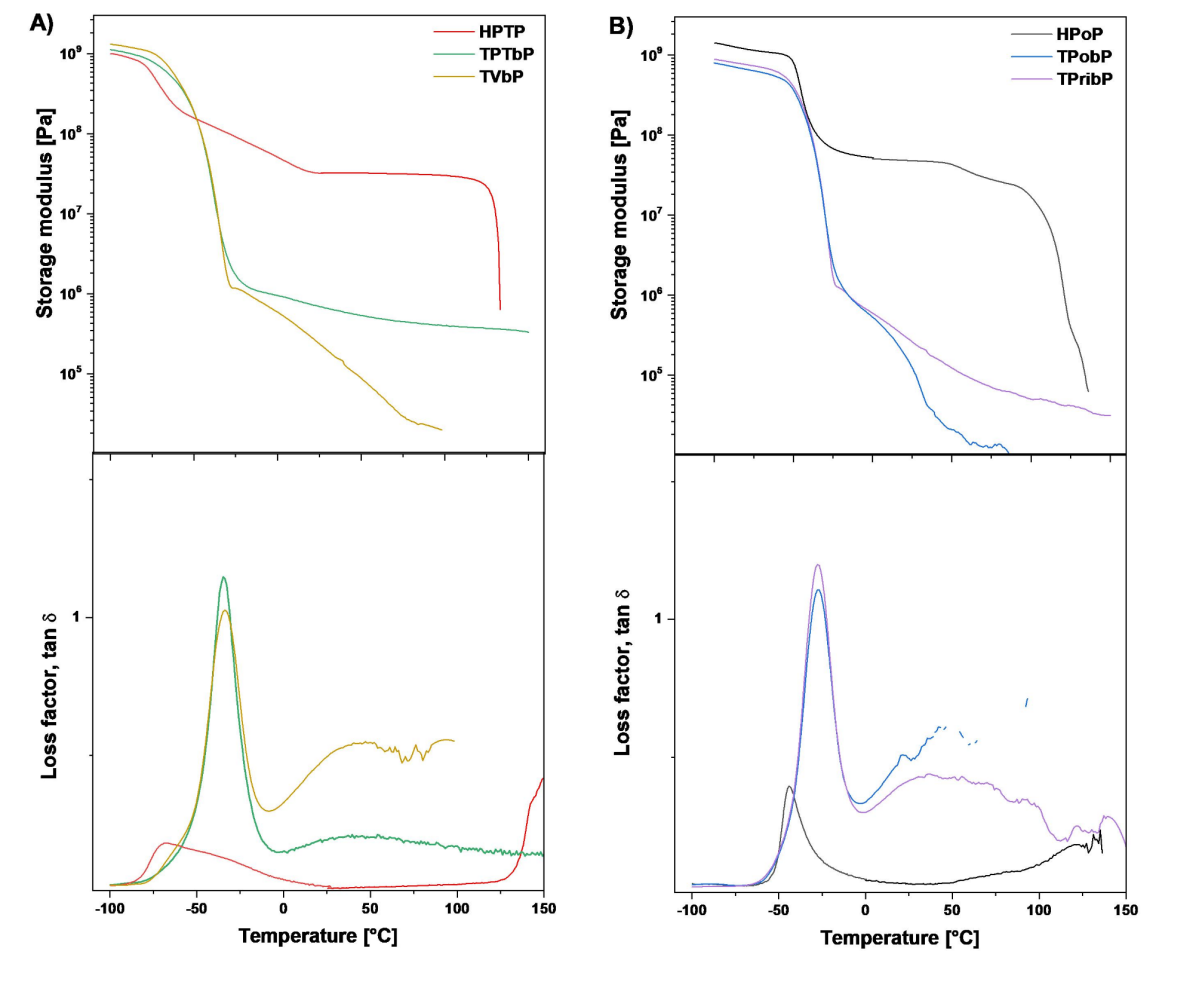
DMTA results of PUs based polyether (**A**) and polyester (**B**) polyols.

The DMTA results show us the supramolecular arrangement of prepared PUs (Fig. 10). The storage modulus curves clearly indicated the main transition in the range of ca. -68 – -28 °C corresponding to the glass transition temperature of SS. From the maximum of the tan delta curves, the main (alpha) temperature (*T*_α_) of SS was determined to be of -34 °C for polyether-based (TPTbP and TVbP) and − 28 °C for polyester-based (TPobP and TPribP) bio-PUs, while the *T*_α_ values of the reference samples based on HDI were slightly lower, -68 °C and − 44 °C for HPTP and HPoP, respectively.

The PU materials based on the bio-based isocyanate Tolonate X FLO 100 were generally more flexible than the materials based on HDI, because in the SS transition region their storage modulus drops sharply by 3 orders of magnitude (from GPa units to MPa units). The subsequent slower decrease of the storage modulus at temperatures above about 0 °C may be related to the melting of crystalline domains, or to Tg of HS. In contrast, the HDI-based PUs were much stiffer materials, as the storage modulus in the transition region of SS only decreased by one order of magnitude. Then there was a gradual decrease of the storage module up to a temperature of about 25 °C, which may be related to the melting of the crystalline phase (see the DSC results above). However, the HDI-derived samples are overall more segregated into SS and HS domains than the bio-based PUs, which was manifested by a significant sharp drop in the storage modulus at temperatures of approx. 120 °C (HPoP) and 130 °C (HPTP), related to the melting of crystalline domains in HS.

## Conclusions

A series of samples containing from 0 wt% to 52 wt% green carbon were prepared. The high green carbon content had no negative effect on the synthesis of the PUs. Samples of both petrochemical and bio-based monomer were prepared using the same method under similar conditions. For bio-based samples, lower curing temperatures could be applied, which is economically beneficial. The structure of the samples obtained was confirmed by FTIR and NMR analysis. The presence of characteristic functional groups and the complete rearrangement of the monomers were confirmed. It was also observed that the materials obtained from petrochemical monomers have a higher degree of phase separation. The SAXS profiles of all PU samples show two distinct peaks, indicating microphase separation and periodic structures in the hard segments. The HDI based samples show peaks at higher Q values, indicating a more compact structure with smaller domain. Bio-PUs were characterized by a reduced intensity of the second peak, suggesting that they have reduced crystallinity or less defined hard segment domains. TG analysis showed that the bio-based PUs were slightly less stable than the reference samples due to their lower initial decomposition temperature. Based on DSC, we confirmed the observations from WAXS, as melting peaks were observed only for the reference samples, confirming their crystalline fraction. However, for the bio-PU samples, glass transition temperatures were observed for the hard and soft segments, depending on the morphology of the samples. The DMTA results show that bio-based polyurethanes are generally more flexible than HDI-based ones, with a sharp decrease in storage modulus in the soft segment (SS) transition region. The *T*_α_ of the SS was higher for polyester-based (-28 °C) than for polyether-based (-34 °C) bio-PUs, while HDI-based samples showed even lower *T*_α_ values. The HDI-based PUs were stiffer, with a more gradual modulus decrease and distinct melting transitions at higher temperatures.

## Data Availability

The datasets used and analysed during the current study available from the corresponding author on reasonable request.
